# Comparison of Montreal cognitive assessment and Mattis dementia rating scale in the preoperative evaluation of subthalamic stimulation in Parkinson’s disease

**DOI:** 10.1371/journal.pone.0265314

**Published:** 2022-04-07

**Authors:** Eileen Gülke, Mohammad Alsalem, Maja Kirsten, Eik Vettorazzi, Chi-un Choe, Ute Hidding, Simone Zittel-Dirks, Carsten Buhmann, Miriam Schaper, Alessandro Gulberti, Christian K. E. Moll, Wolfgang Hamel, Johannes Koeppen, Christian Gerloff, Monika Pötter-Nerger

**Affiliations:** 1 Department of Neurology, University Medical Center Hamburg-Eppendorf, Hamburg, Germany; 2 Institute of Medical Biometry and Epidemiology, Center for Experimental Medicine, University Medical Center Hamburg-Eppendorf, Hamburg, Germany; 3 Department of Neurosurgery, University Medical Center Hamburg-Eppendorf, Hamburg, Germany; 4 Department of Neurophysiology and Pathophysiology, University Medical Center Hamburg-Eppendorf, Hamburg, Germany; Oslo Universitetssykehus, NORWAY

## Abstract

**Introduction:**

The preoperative evaluation of Parkinson’s Disease (PD) patients for subthalamic nucleus deep brain stimulation (STN-DBS) includes the assessment of the neuropsychological status of the patient. A widely used preoperative test is the Mattis Dementia rating scale (MDRS). However, the Montreal cognitive assessment (MoCA) has also been proven to be a sensitive, time-sparing tool with high diagnostic validity in PD. We evaluate the utility of the MoCA as a preoperative screening test for PD patients undergoing bilateral STN-DBS.

**Methods:**

In this single-centre, retrospective study, we analysed pre- and postoperative assessments of MoCA, MDRS, Movement disorder society-Unified PD Rating Scale-motor examination, PD Questionnaire-39 and levodopa equivalent daily dose. Longitudinal outcome changes were analysed using paired t-test, Pearson’s correlation coefficient, linear regression and CHAID (chi-square automatic interaction detector) regression tree model.

**Results:**

Clinical motor and cognitive scores of 59 patients (61.05±7.73 years, 24 females) were analysed. The MoCA, but not the MDRS, identified significant postoperative cognitive decline in PD patients undergoing STN-DBS. The preoperative MoCA score correlated with postoperative quality of life improvement, whereas the MDRS did not. PD patients with a MoCA score ≤ 23 points had a significant decline of quality of life after DBS surgery compared to patients > 23 points.

**Conclusion:**

This study identifies the MoCA as an alternative test within the preoperative evaluation of PD patients for the detection of neuropsychological deficits and prediction of the postoperative improvement of quality of life.

## Introduction

In Parkinson´s disease (PD), deep brain stimulation (DBS) of the subthalamic nucleus (STN) has emerged as an effective, established therapy in specific patients in advanced stages of the disease [1]. Careful patient selection is an essential determinant of consistent, favourable, postoperative outcomes after DBS surgery [2], since about one third of DBS failures could be attributed to inappropriate patient selection in past studies [3] highlighting the need for careful preoperative evaluation [2]. The current recommendations for appropriate, preoperative patient screening comprise the assessment of the overall health condition, age, comorbidities, neuropsychiatric profile, the severity and L-dopa responsiveness of the different motor symptoms and motor fluctuations [2]. One important aspect in the decision process represents the patient´s cognitive profile and the risk to develop postoperative cognitive decline, since new postoperative onset of cognitive disturbances due to STN-DBS affects activities of daily living and psychiatric function [4].

Studies on cognitive performances in STN-DBS operated PD patients revealed heterogeneous results. Recurrent findings are unchanged total scores of screening tests assessing global cognitive function in PD patients with STN-DBS, but deterioration of specific cognitive subdomains. One of the most often used screening tests represents the Mattis Dementia Rating Scale (MDRS) [5]. The MDRS is recommended by the Movement disorder society (MDS) to detect mild cognitive impairment (MCI) in Parkinson’s disease [6]. Multicenter, randomised, controlled trials revealed no change of global cognitive function in the DBS-group postoperatively compared to PD patients in the non-surgical group as revealed by an unchanged total score of the MDRS [7, 8]. However, further specified neuropsychological tests showed an impairment of executive functions as decline of verbal fluency and worsened attentional inhibition of automated reactions in PD patients with STN-DBS [9]. Thus, STN-DBS tends to impair specific cognitive subdomains, which are not adequately measured by the total score of the MDRS.

The MoCA has become a popular cognitive screening instrument in PD addressing particular frontal and executive functioning [6, 10]. The test has been shown to be more sensitive with equivalent specificity compared to the MDRS [11] in a general PD patient population. To date, the MoCA has not been established as a standard tool in the preoperative DBS evaluation procedure. We hypothesized that 1. the MoCA is a favourable perioperative, cognitive screening test to identify patient’s cognitive status, as frontal executive dysfunction is better addressed in shorter application time 2. the preoperative MoCA can predict changes in quality of life (QoL).

## Methods

### Study design and ethical approval

The study was performed as a single-centre, retrospective study in STN-DBS operated PD patients. Patients were recruited between February 2014 and October 2018 and were invited to preoperative baseline and postoperative follow-up visits. The study was conducted in agreement with the Code of Ethics of the World Medical Association (Declaration of Helsinki, 2018). Ethical approval was obtained at the local ethics comitee “Ethik-Kommission der Ärztekammer Hamburg” (PV 5281) and written informed consent was obtained.

### Participants

Preoperatively, all PD patients were screened and selected in accordance to international guidelines of DBS surgery (CAPSIT protocol) [12]. Patients required a significant improvement of the motor-subscore of the Movement Disorder Society Unified Parkinson’s Disease Rating Scale (MDS-UPDRS III) after intake of immediate-release soluble levodopa. PD patients were studied if 1. Parkinson´s disease in Hoehn & Yahr 2–3 was present 2. bilateral DBS electrodes were implanted in the STN either under local or general anesthesia with microelectrode recordings for optimized targeting 3. a stable postoperative condition was ensured (approximately 6 months postoperatively) and 4. sufficient German language skills were present. Exclusion criteria comprised patients with atypical Parkinsonian syndromes or any alternative cause of parkinsonism. Additionally, patients with severe preoperative dementia (MDRS < 130), or unstable neuropsychiatric symptoms were not considered for DBS surgery. PD subtyps were retrieved from corresponding medical reports and were clinically assessed by the clinician in charge.

### Surgical procedure

For surgery, a Zamorano-Dujovny frame (Stryker Leibinger, Freiburg, Germany) was mounted on the patient’s head. Stereotactic image-based target localisation was performed by fusion of gadolinium-enhanced volumetric T1 and T2 weighted MRI sequences and computerized tomography (iPlan, BrainLAB Inc., Westchester, IL, USA). After determination of the anterior and posterior commissure (AC-PC line), the dorsal STN was targeted 11–12 mm lateral to midline, 0–3 mm posterior to the midcommissural point, and 1–3 mm inferior to the intercommissural plane on both sides. The access path was localised at the coronal suture. Final electrode placement was guided by intraoperative microelectrode recording (MER) by use of three parallel tracks to map the subthalamic region with tungsten electrodes (NeuroProbe electrodes, Alpha Omega Inc., Nazareth, Israel, BenGun configuration). The optimal target site for electrode implantation was determined by MERs and clinical evaluation of macrostimulation responses.

### Clinical assessment and questionnaires

Pre- and postoperative motor assessments were performed both in the off-medication state after overnight withdrawal of medication (MED OFF) and in the on-medication state after medication intake with supramaximal dose of 1.5x morning dosage (MED ON). Postoperative tests were performed with stimulation ON (STIM ON). The levodopa equivalent daily dose (LEDD) was computed according to the method by Schade et al. [13]. The neuropsychological tests MDRS and MocA were performed within the same patients.

The 38-item MDRS comprises five scales measuring different cognitive subdomains (attention, initiation and perseveration, construction, conceptualization, memory impairment). The items are presented in a hierarchical order, meaning that a correct answer to the first most difficult item of a subscale allows the examiner to give credits for the subsequent items [14]. The maximum total score is 144. It takes approximately 20–30 minutes to perform the MDRS. For Parkinson’s disease-mild cognitive impairment (PD-MCI) a cut-off score of ≤ 137 points is proposed [15]. The Montreal Cognitive Assessment (MoCA) is a one-page test administered in approximately 10 minutes with a maximum score of 30 points. The MoCA consists of subtests assessing visuospatial abilities (clock-drawing task, three dimensional cube copy), short-term memory (learning trials of five nouns and delayed recall test), executive functions (trail making B test, phonemic fluency test, verbal abstraction test), attention and concentration (attention task, serial substraction task, digits forward and backward counting), language (naming task, repetition of two syntactically complex sentences, verbal fluency task) and orientation (time and place) [10]. As the MoCA has been translated into fifty-two different languages, it serves as a cross-cultural screening tool, although adjustments for education and cultural background might be necessary [6]. Two German versions of the test were used in random order. For PD-MCI, a cut-off score of ≤ 26 points is suggested [16].

Both neuropsychiatric tests MocA and MDRS were pre- and postoperatively performed in the same patients in random order in the regular on-medication state in an one-to-one interview on 2 separate days during stationary visits to avoid mental exhaustion. PD patients scoring lower than 137 points in the MDRS and lower than 26 points in the MoCA test preoperatively underwent extensive neuropsychological assessments including the Consortium to Establish a Registry for Alzheimer’s Disease neuropsychological battery (CERAD). All PD patients are discussed in an interdisciplinary DBS board taking into account the patient’s overall cognitive performance during the hospital stay, medical history and health condition. Borderline cognition is extensively discussed and in doubt, a follow-up visit performed.

To evaluate QoL, the PD Questionnaire-39 (PDQ-39) was applied [17]. The PDQ-39 is a self-report questionnaire consisting of 39 items to assess frequent, specific, health-related problems within the last month. PDQ-39 comprises 8 different quality of life dimensions: mobility, activities of daily living, emotional well-being, stigma, social support, cognition, communication and bodily discomfort. The Parkinson’s disease summary index (PDQ-39 SI) provides a global measure of the subjectively perceived health status in PD patients [17]. The SI is derived by the sum of the calculated percentages of the eight PDQ-39 subscores divided by eight (the number of subscales) resulting in a score between 0 and 100 (higher percentages indicating impaired quality of life).

### Statistics

Statistical analysis was performed using SPSS V 27.0 (IBM Corporation). Changes of pre- and postoperative scores were analysed by Pearson’s correlation coefficient, paired t-tests and linear regression. They are presented as mean±standard deviation of the mean (SD). Level of significance was set p ≤ 0.05. A conditional inference tree model was performed both for MoCA and MDRS using chi-square automatic interaction detector (CHAID). Statistical analysis was only performed for full datasets when preoperative MDRS, MoCA and pre- and postoperative PDQ-39 SI were applicable.

## Results

### Baseline characteristics

Of 84 PD patients in our database, 59 patients (61.1±7.7 years, 24 females) suffering from advanced idiopathic PD were retrospectively included. Preoperative DBS evaluation was performed 3.84± 2.85 months before surgery. 7 patients were classified as tremor-dominant PD subtypes, 31 patients as akinetic-rigid subtypes and 17 patients as intermediate subtypes. 2 PD patients were classified as young-onset Parkinsonism. Disease duration was 10.37±4.30 years, preoperative levodopa equivalent daily dose (LEDD) was 1171.62±543.44mg.

### Postoperative changes of clinical scores in PD patients with STN-DBS

Postoperative follow-up visits were performed 6.11±2.00 months after surgery based on regular follow up appointments (Table 1, S1 Table).

**Table 1:** Preoperative and postoperative PD patients scores.

	Preoperative			Postoperative		
	n	M	SD	n	M	SD
**MoCA**	54	27.22	2.19	54	26.43	3.17
**MDRS**	43	140.42	2.99	43	140.02	5.01
**PDQ-39 SI**	59	29.20	14.12	59	21.41	12.86
**MDS-UPDRS III MED OFF**	54	36.72	17.86	54	26.41	13.24
**MDS-UPDRS III MED ON**	58	16.53	10.80	56	16.54	9.42

MoCA, Montreal cognitive assessment; MDRS, Mattis dementia rating scale; PDQ-39 SI, Parkinson’s Disease Questionaire-39 Summary Index; MDS-UPDRS III, Movement Disorder Society Unified Parkinson’s Disease Rating Scale; MED OFF, off-medication state; MED ON, on-medication state; M, mean; SD, standard deviation of mean. Postoperative tests were performed with DBS on.

**Fig 1:**
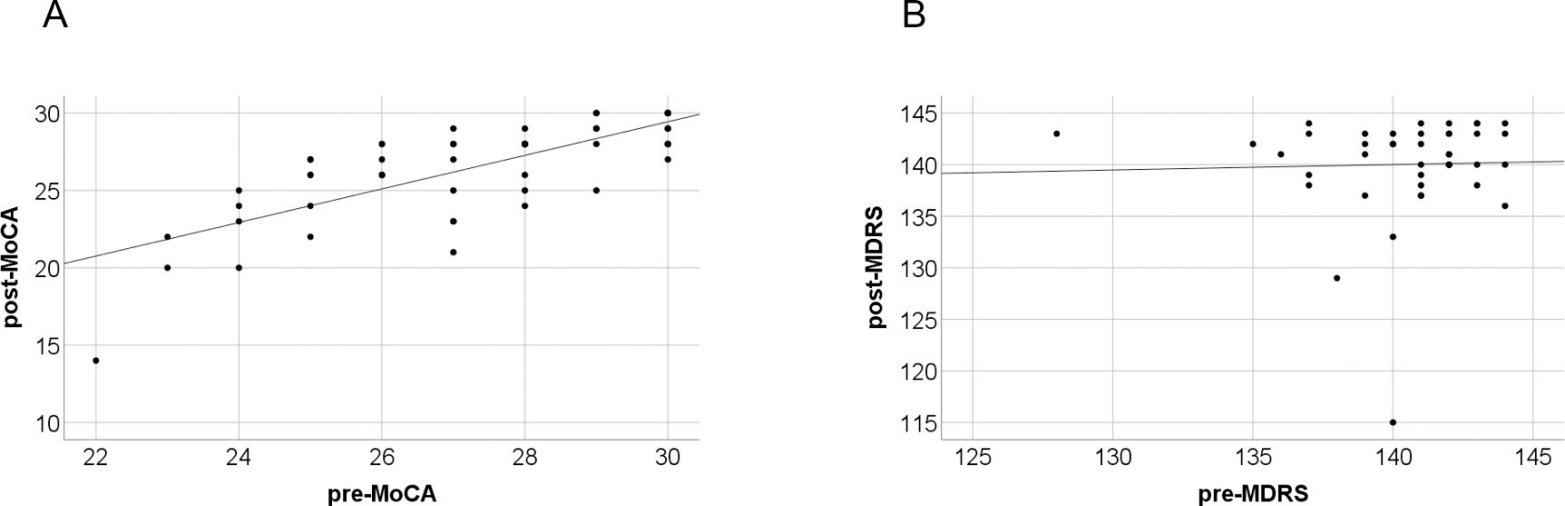
Linear regression of pre- and postoperative MoCA and MDRS. A: Scatterplot of pre- and post-MoCA scores with significant linear regression slope of 1.09 points (p<0.001). B: Scatterplot of pre- and post-MDRS scores with no significant linear relationship between pre-and post-MDRS scores (p = 0.843). pre: preoperative; post: postoperative.

Postoperative LEDD was 732.38±395.10mg. In accordance with previous studies, motor symptoms and quality of life improved after STN-DBS in the observed PD patient cohort. The preoperative MDS-UPDRS III score in MED OFF condition (36.72±17.86) improved to 26.41±13.24 in MED OFF/STIM ON condition (p<0.001). The preoperative PDQ-39 SI (29.20±14.12) was reduced to 21.41±12.86 points postoperatively (p<0.001) indicating a significant improvement of quality of life in the total cohort of PD patients. Cognitive performances of PD patients pre- and postoperatively after STN-DBS differed depending on the cognitive screening test. The preoperative MoCA score was 27.22±2.19, the postoperative MoCA score was significantly reduced to 26.43±3.17 (p = 0.008). A correlation was found between the preoperative MoCA and the postoperative MoCA (Pearson Correlation coefficient r = 0.748, p = 0.01). A linear regression revealed a linear relationship (regression coefficient = 1.09, p<0.001, R^2^ = 0.559) (Fig 1A). In contrast, the preoperative MDRS score (140.42±2.99) remained stable (140.02±5.01) postoperatively (p = 0.654). There was no correlation between pre- and postoperative MDRS (r = 0.03) and no linear dependance (regression coefficient = 0.05, p = 0.843, R^2^ = 0.001, Fig 1B).

**Fig 2:**
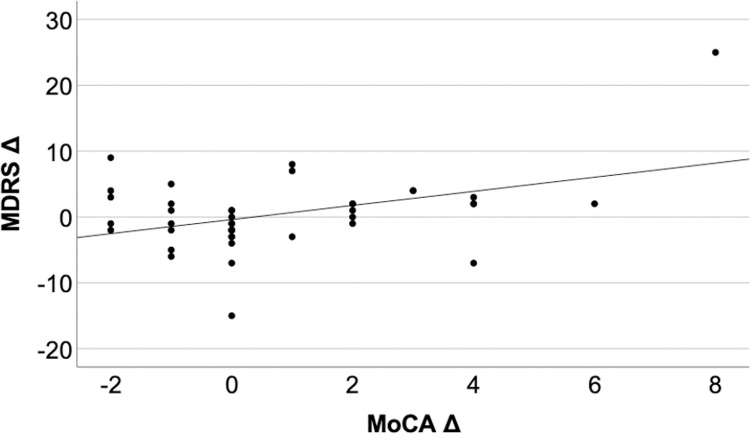
Linear regression of pre- and postoperative changes of MoCA and MDRS. Scatterplot of Δ MoCA and Δ MDRS as dependent variable with linear regression slope of 1.07 points (p = 0.006) but weak model prediction. MoCA Δ, preoperative-postoperative MoCA; MDRS Δ, preoperative-postoperative MoCA.

There was a significant change of the MoCA score pre- to postoperatively (MoCA Δ 0.80±2.11) within 69.5% of the operated PD patients. The MDRS was not significantly changed perioperatively (MDRS Δ 0.40±5.75 points). There was a slight, but significant linear correlation between Δ MocA and Δ MDRS (r = 0.414, p = 0.01, regression coefficient = 1.07, p = 0.006, R2 = 0.273, Fig 2).

Patients with a larger postoperative cognitive decline with MoCa Δ ≥ 3 (n = 11) were older, had longer disease duration and higher preoperative LEDD at the time of surgery, and less LEDD reduction postoperatively compared to the rest of the cohort (S2 Table). Additionally, the postoperative PDQ-39 SI was higher, indicating a lower quality of life.

### Prediction of postoperative quality of life

**Fig 3:**
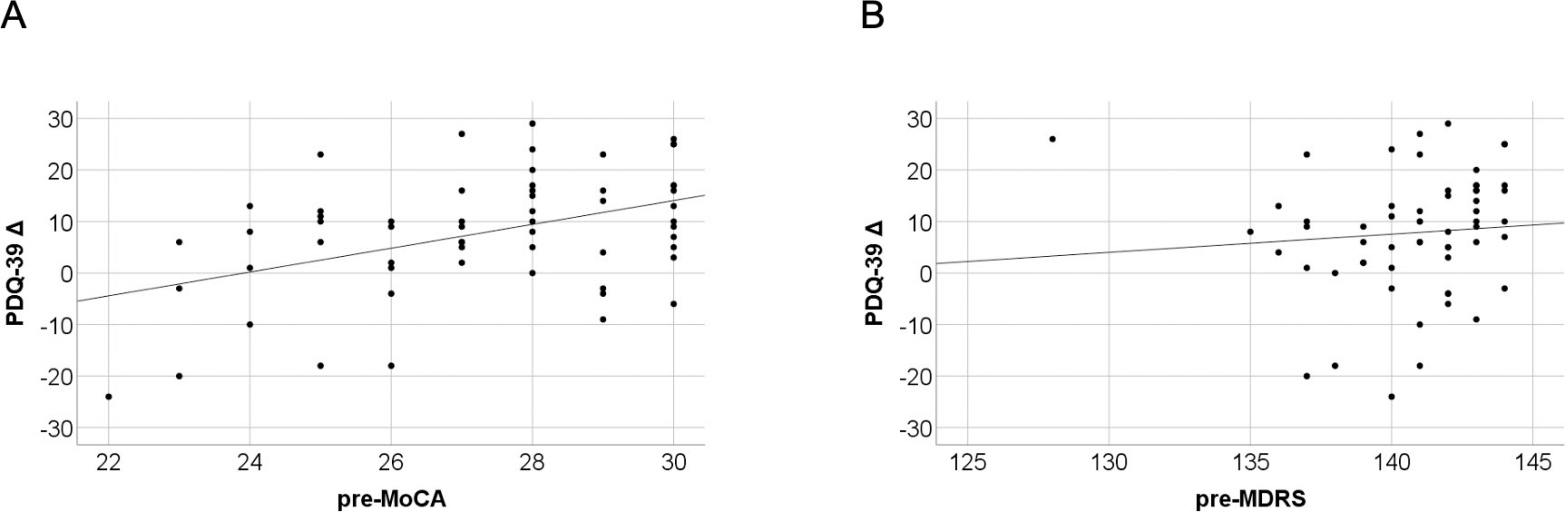
Linear regression of preoperative MoCA, MDRS and perioperative PDQ-39 SI changes. A: Scatterplot of pre-MoCA and Δ PDQ-39 with significant linear regression slope of 2.31 points (p = 0.001). Pre-MoCA predicted postoperative quality of life. B: Scatterplot of pre-MDRS and Δ PDQ-39. There was no significant linear relationship between pre-MDRS and Δ PDQ-39 (p = 0.51). pre: preoperative; Δ PDQ-39: difference of preoperative PDQ-39 SI minus postoperative PDQ-39 SI.

Linear regression analysis was performed to evaluate potential preoperative predictors of postoperative changes of quality of life and cognitive performances. A linear relationship was found between the preoperative MoCA and the change of pre- to postoperative PDQ-39 SI (Δ PDQ-39) (regression coefficient = 2.31, R^2^ = 0.192, p = 0.001) (Fig 3A). Hence, the higher the preoperative MoCA score of the patient, the larger the postoperative improvement of QoL. No dependance was seen between preoperative MDRS and Δ PDQ-39 (regression coefficient = 0.35, 95%-CI[-0.72;1.43], R^2^ = 0.008, p = 0.51) (Fig 3B).

### Preoperative MoCA as predictor of postoperative quality of life

**Fig 4:**
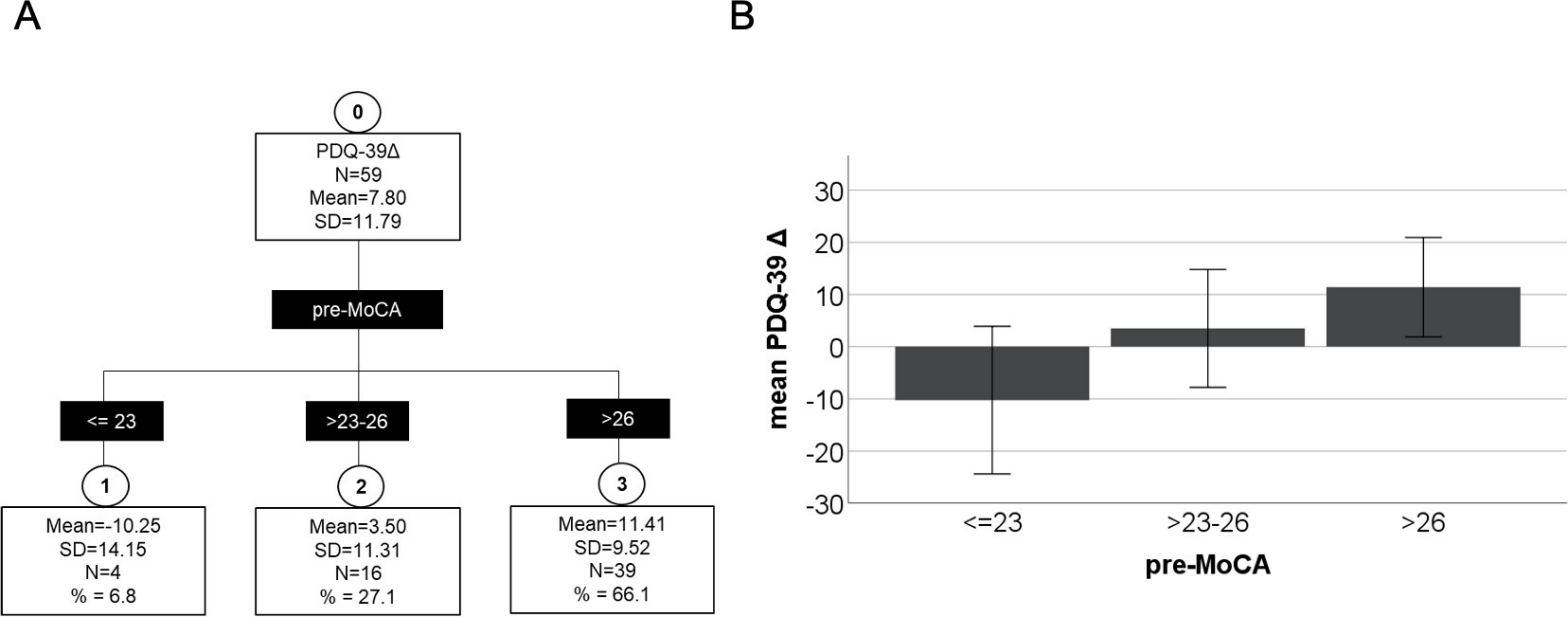
Conditional inference tree of preoperative MoCA and perioperative PDQ-39 SI changes. A: Conditional inference tree revealed a three-fold partitioning of patients by the pre-MoCA score. Patients scoring ≤ 23 in the pre-MoCA had a decline of post-PDQ-39 SI. Pre-MoCA scores 23–26 resulted in a slight, but significant improvement of post-PDQ-39 SI. Patients >26 points showed the most reliable, positive post-PDQ-39 SI improvement. B: A pre-MoCA >23 points resulted in an improvement of quality of life. Patients with a pre-MoCA < = 23 showed a postoperative decline of quality of life. Pre: preoperative; post: postoperative; SD = standard deviation; N = number; % = percentage of patients; mean = Δ PDQ-39 mean; Δ PDQ-39 = difference of preoperative PDQ-39 SI minus postoperative PDQ-39 SI; Error bar = ± 1 standard deviations.

We used a chaid (chi-square automatic interaction detector) regression tree model based on the change of PDQ-39 SI (Δ PDQ-39) as the target variable and the preoperative MoCA as its predictor to screen for cut-off values to differentiate groups of patients profiting from DBS surgery in terms of QoL. At each step, CHAID chooses the independent variable that has the strongest interaction with the dependent variable. Categories of each predictors are merged if they are not significantly different with respect to the dependent variable. No conditions were found for the preoperative MDRS score. In contrast, a threefold cTree was established for the preoperative MoCA Score (p< 0.001) (Fig 4A). Patients scoring higher than 26 (n = 39) in the preoperative MoCA had a mean improvement of 11.41±9.52 Δ PDQ-39. A score ≤ 23 (n = 4) predicted a significant decline of QoL after DBS surgery (-10.25±14.15 Δ PDQ-39). Patients scoring >23–26 (n = 16) in the preoperative MoCA showed a moderate but significant improvement of the ΔPDQ-39 (3.50±11.31). Thus, PD Patients with a preoperative MoCA score ≥ 23 profited from STN-DBS in terms of QoL (Fig 4B).

Comparing the detected MoCA subgroups, it is evident that PD patients with higher preoperative PDQ-39 and thus lower quality of life profited the most from STN DBS surgery (Table 2).

**Table 2:** Comparison of patient characteristics in relation to MoCA performance.

pre-MoCA		≤23	23–26	>26
**male**	**n**	2	10	23
**female**	**n**	2	6	16
**age at surgery**	**M**	66.75	60.69	60.62
**disease duration**	**M**	10.75	10.00	10.49
**first symptoms onset**	**M**	12.75	10.07	11.24
**Hoehn & Yahr**	**M**	2.25	2.27	2.35
**pre-LEDD**	**M**	815.75	1106.30	1234.92
**post-LEDD**	**M**	699.44	851.02	687.09
**pre-MDS-UPDRS III MED OFF**	**M**	25.75	40.87	35.97
**pre-MDS-UPDRS III MED ON**	**M**	6.75	18.80	16.67
**post-MDS-UPDRS III MED OFF**	**M**	19.50	30.47	25.89
**post-MDS-UPDRS III MED ON**	**M**	12.25	16.60	16.97
**pre-PDQ-39 SI**	**M**	16.75	27.88	31.03
**post-PDQ-39 SI**	**M**	27.00	24.38	19.62

MoCA, Montreal cognitive assessment; MDRS, Mattis dementia rating scale; PDQ-39 SI, Parkinson’s Disease Questionaire-39 Summary Index; MDS-UPDRS III, Movement Disorder Society Unified Parkinson’s Disease Rating Scale; MED OFF, off-medication state; MED ON, on-medication state; M, mean; Postoperative tests were performed with DBS on.

Patients scoring higher than 26 in the preoperative MoCA (n = 39) were younger (60.62±7.43), had higher preoperative LEDD (1234.92±592.46), had the highest preoperative PDQ-39 SI (31.03±15.00) and MDS-UPDRS III MED OFF (35.97±13.33) of the three groups. Within this group, 23.1% had general anesthesia, 71.8.% underwent DBS surgery under local anesthesia and 5.1% were switched intraoperatively from local anesthesia to general anesthesia.

Patients with preoperative ≤ 23 MoCA (n = 4) were older (66.75±2.75), had a lower preoperative LEDD (815.75±148.18) and were less severly affected with MDS-UPDRS III MED OFF (25.75±8.54) and MDS-UPDRS III MED ON (6.75±2.63). 50% were operated in general anesthesia, the other 50% in local anesthesia. The PDQ-39 SI worsened from preoperative 16.75±6.19 to postoperative 27.00±10.10 indicating lower quality of life.”

Patients with Δ MoCa ≥ 3 (n = 11) were older at surgery (62.45±8.19), had longer disease duration (11.45±3.98) and their preoperative LEDD (12.81.05±806.55) was higher compared to the rest of the cohort (1090.59±687.67) (S2 Table). Moreover, the postoperative LEDD (806.55±384.79) was less reduced in comparison to the rest of the analyzed patients (687.67±370.68). Additionally, the postoperative PDQ-39 SI (24.45±12.95) was higher, indicating a lower quality of life.

## Discussion

In this single-center, retrospective analysis of PD patients with STN-DBS, we identified the MoCA as an adequate cognitive test to assess preoperative cognitive function and potential postoperative cognitive decline. The MoCA but not the MDRS detected subtle cognitive postoperative changes. Moreover, the preoperative MoCA was a predictor for postoperative changes of quality of life. A preoperative MoCA score ≤23 was associated with postoperative worsening of quality of life whereas a score >23 with a significant improvement of quality of life.

To date, the most commonly used preoperative cognitive screening test is the MDRS as part of the CAPSIT protocol [12]. The MDRS provides several advantages compared to other cognitive scales. First, it is a recommended cognitive rating scale with fine clinimetric properties and normative data as well as cut-offs for dementia (≤ 132) [14] and MCI (≤137) [6, 15]. A cut-off score of ≤ 120 represents a discriminatory value for the diagnosis of Parkinson’s disease dementia (PDD) [18]. Second, the MDRS comprises cognitive subscales to administer specific, different cognitive domains, especially executive and attentional function [11]. This is relevant since the executive domain is particularly impaired in PD patients [19]. Third, in one multicenter, randomised study, the preoperative MDRS score was a predictor for potential failure of postoperative improvement of Qol [20]. A borderline preoperative MDRS score was associated with a limited Qol measured by the Parkinson’s disease questionnaire PDQ-39 [20]. However, a recent study revealed the opposite and found no correlation between the MDRS and postoperative changes of QoL [21]. This is in line with our findings that the MDRS score did not correlate with postoperative changes of PDQ-39 SI. Moreover, the lengthy time to administer the MDRS is a challenge in clinical practice [6]. Since previous DBS-trials demonstrated that in the specific PD-DBS cohort the MDRS total score is not sensitive to capture subtle changes, the MoCA might be a better cognitive screening test to predict and assess DBS-associated changes in the cognitive domain. The MoCA takes only approximately 10 minutes and provides a higher sensitivity in all subsections, especially in the visuospatial and memory subdomain compared to the MDRS [11]. Thus, the MoCA seems to represent a suitable test to asses the cognitive status in PD, since PD patients show particularly deficits in executive function, attention, visuospatial skills and memory [6]. For PD-MCI, a cut-off score of ≤ 26 points is suggested [16]. However, there is an ongoing debate about the adequate MoCA cut-off values. In the original validation study of the MoCA, there was a proposed cut-off value of 26 to differentiate healthy controls from patients with mild cognitive impairment [10]. However further exploration revealed the possibility of incorrect, false positive diagnosis with low specificity with that cut-off value of 26 [22]. In contrast, a MoCA cut-off below 23–23.5 was proposed for the diagnosis of mild cognitive impairment [23]. This fits with our finding that scores ≤ 23 appear to be predictive for a decline in quality of life after STN-DBS surgery. We found a subtle, but significant postoperative cognitive decline in PD patients with STN-DBS using the MoCA. There are previous results concerning the longitudinal change in MoCA score in a general population of PD patients. In a 30-month prospective study of non-surgical PD patients, the MoCA decreased by 2.56 points [24] whereas in another surgical PD patient cohort, the MoCA remained stable after 6 months [25]. In the latter study, PD patients with preoperative MoCA> 25 participated. Our cohort included patients with partially lower preoperative MoCA scores, which might account for the substantial decline in cognitive performance.

In the interpretation of these findings one needs to consider that the potential postoperative, cognitive impairment is due to several independent factors. On the one hand, there is a gradual, cognitive decline due to the disease progression in PD patients and age in the long-term postoperative course. As shown in a previous meta-analysis of initially non-demented PD patients without any DBS surgery, there was a significant decline in global cognitive ability after 2–3 years, particularly in the subdomains visuoconstructive skills and memory [26]. The preoperative PD patient profile seems to be interrelated with potential postoperative decline. Preoperative impaired attention, higher anti-parkinsonian medication, higher axial scores, lower L-Dopa responsiveness were shown to be correlated with worse cognitive postoperative performances after STN-DBS indicating a particular vulnerability of specific PD patient cohorts [27]. On the other hand, short-term postoperative cognitive decline might also be associated with changes of the postoperative medication. After STN-DBS, dopaminergic medication is reduced by about 50%. The postoperative reduction of dopaminergic medication might therefore result in drug withdrawal phenomena with bradyphrenic worsening [28]. STN-DBS can impact cognitive performance as demonstrated in acute DBS “ON-OFF” studies. Assessment of STN-DBS patients with DBS switched “ON” and “OFF” on one day revealed short-term specific effects as improved cognitive flexibility but increased error rates in interference or response inhibition tasks [29]. Also the surgical procedure itself might hamper cognition by the electrode trajectory affecting cortico-subcortical structures [30]. Evaluation of the varying cortical lead entry points, subcortical electrode paths and positions of the active electrode contacts in STN-DBS patients revealed an increased risk of global cognitive disturbances and cutback of working memory in relation to the amount of caudate head volume affected by the electrode trajectories [30]. Therefore, potential cognitive decline after DBS surgery is a complex problem due to the DBS surgery procedure itself, potential DBS stimulation effects, medication, disease progression and patients profile. These different factors might add up resulting in the “total amount of cognitive decline”.

There are limitations of this study. The sample size is small for a comparative study but nevertheless, we found evidence of the utility of the MoCA for outcome prediction. MoCA as well as MDRS are screening instruments and cannot replace proper neuropsychological evaluation in case of borderline cognitive function. We analysed retrospective, clinical routine data. PD subtypes were retrieved from medical records, which were performed by the clinician in charge. These “real world data” are heterogeneous with potential outliers. As an example, one patient worsened from the normal range MDRS score of 140 points to 115 points postoperatively indicating considerable cognitive decline. Remarkably, this patient already would have been considered as demented according to the preoperative Moca score of 22 points, with further decline to 14 points postoperatively. This individual observation supports the hypothesis that the preoperative MoCA <23 is a more sensitive tool to predict postoperative further cognitive decline than the MDRS. Consequently, patients with low preoperative MoCA scores should be excluded from STN DBS surgery in terms of postoperative cognitive decline and reduction of quality of life.

In clinical practice, we adhere to specific practice guidelines as neuropsychological testing in regular on-medication state. However, in fluctuating patients, it cannot be excluded that a patient was tested in the beginning of a wearing-off phase leading to overestimation or false classification of cognitive deficits.

### Conclusion

In summary, these findings identify the MoCA as an adequate preoperative cognitive screening test of the patient’s overall cognitive function for DBS evaluation. The MoCA detects potential postoperative cognitive decline and enables the prediction of postoperative changes of quality of life. Further validation of the MoCA test in a prospective, larger, multicenter study should be performed in DBS patients and compared with a neuropsychological test battery as point of reference.

## Supporting information

S1 Table Individual pre- and postoperative scores of MoCA, MDRS and PDQ-39 SI. Pre, preoperative; post, postoperative; MoCA, Montreal cognitive assessment; MDRS, Mattis dementia rating scale; PDQ-39 SI, Parkinson’s Disease Questionaire-39 Summary Index; Pre- and postoperative tests were performed in on-medication state. Postoperative tests were performed with DBS on.
(TIF)

S2 Table Patient’s characteristics with ≥3 MoCA Δ. Age at surgery, disease duration and first symptoms onset given in years. MoCA Δ, preoperative MoCA-postoperative MoCA; MoCA, Montreal cognitive assessment; MDRS, Mattis dementia rating scale; PDQ-39 SI, Parkinson’s Disease Questionaire-39 Summary Index; MDS-UPDRS III, Movement Disorder Society Unified Parkinson’s Disease Rating Scale; MED OFF, off-medication state; MED ON, on-medication state; M, mean; Postoperative tests were performed with DBS on.
(TIF)
