# Supplementary figures and images for: Comparison of Montreal cognitive assessment and Mattis dementia rating scale in the preoperative evaluation of subthalamic stimulation in Parkinson’s disease
Source: PLoS One. 2022 Apr 7;17(4):e0265314. doi: 10.1371/journal.pone.0265314 (PMC8989318; doi:10.1371/journal.pone.0265314)

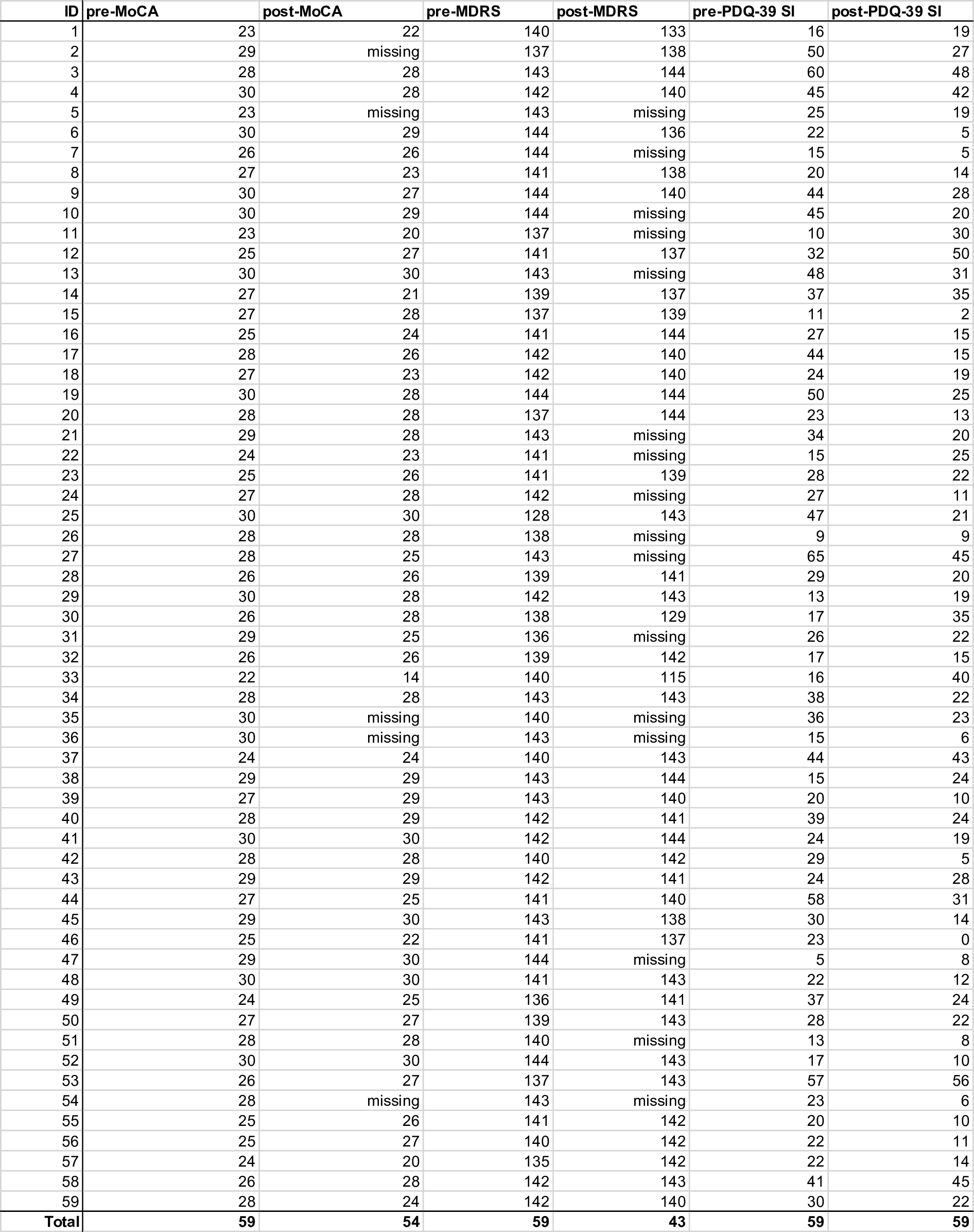

Supplement: S1 Table — Pre, preoperative; post, postoperative; MoCA, Montreal cognitive assessment; MDRS, Mattis dementia rating scale; PDQ-39 SI, Parkinson’s Disease Questionaire-39 Summary Index; Pre- and postoperative tests were performed in on-medication state. Postoperative tests were performed with DBS on. (TIF) [file pone.0265314.s001.tif]

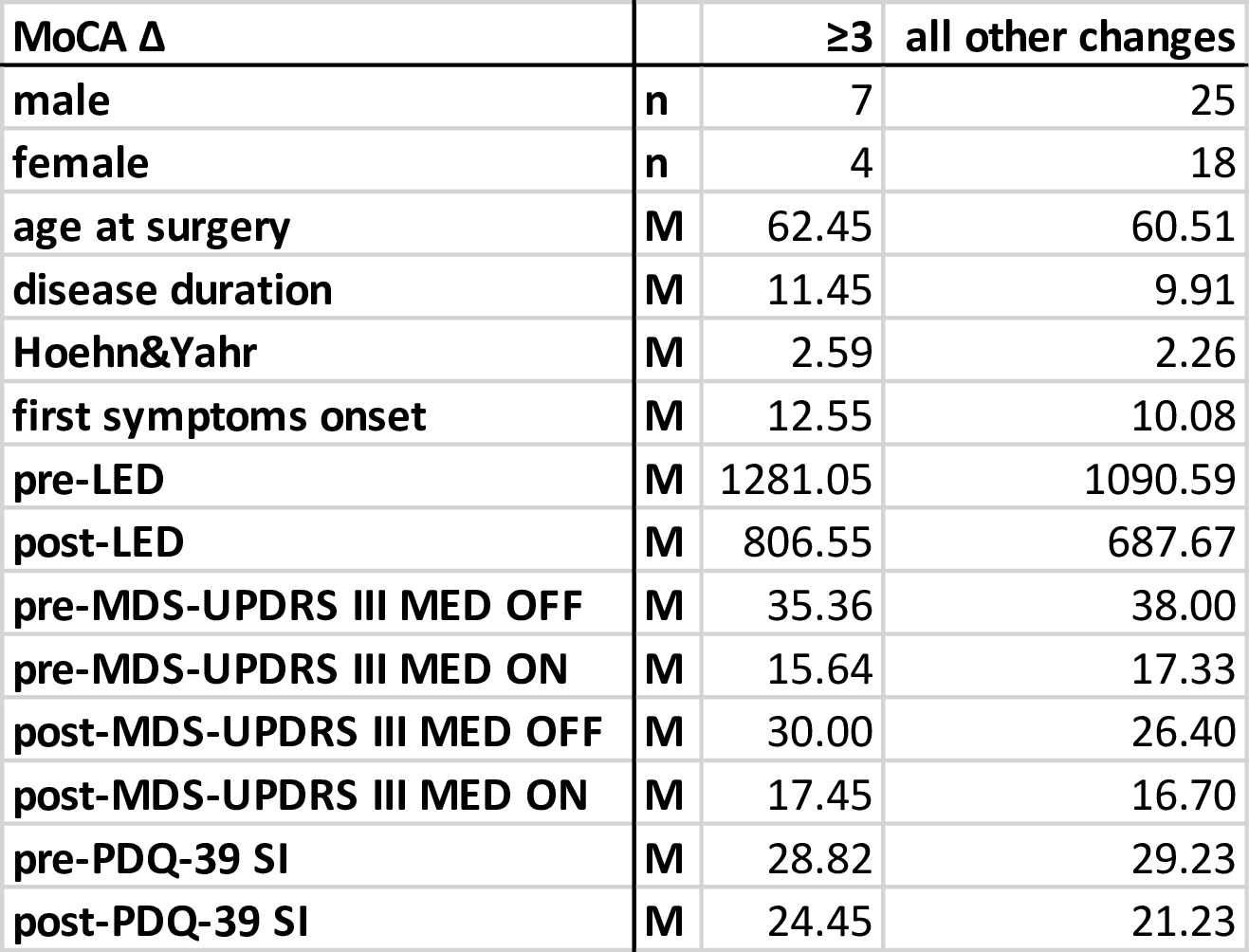

Supplement: S2 Table — Age at surgery, disease duration and first symptoms onset given in years. MoCA Δ, preoperative MoCA-postoperative MoCA; MoCA, Montreal cognitive assessment; MDRS, Mattis dementia rating scale; PDQ-39 SI, Parkinson’s Disease Questionaire-39 Summary Index; MDS-UPDRS III, Movement Disorder Society Unified Parkinson’s Disease Rating Scale; MED OFF, off-medication state; MED ON, on-medication state; M, mean; Postoperative tests were performed with DBS on. (TIF) [file pone.0265314.s002.tif]
